# Glycopyrrolate and Post-Operative Urinary Retention: A Narrative Review

**DOI:** 10.7759/cureus.11379

**Published:** 2020-11-08

**Authors:** Jason Low, Mauricio Escobar, Sebastian Baquero, Howard S Goldman, Gerald Rosen

**Affiliations:** 1 Anesthesiology, Herbert Wertheim College of Medicine of Florida International University, Miami, USA; 2 Anesthesiology, Mount Sinai Medical Center of Florida, Miami Beach, USA

**Keywords:** glycopyrrolate, urinary retention, economic impact, operative planning, risk factors

## Abstract

The use of non-depolarizing neuromuscular blockade (NDNMB) necessitates the use of reversal agents. Glycopyrrolate, an anticholinergic agent, is commonly used in combination with neostigmine, an anticholinesterase, for the reversal of neuromuscular blockade medications. Glycopyrrolate is known to effect on the genitourinary system adversely with an inhibitory effect on bladder contraction, bladder hypotonia, and increase in the frequency of urinary retention. Many studies analyzing the association between glycopyrrolate and urinary retention are outdated and published over a decade ago. The decade old studies were retrospective and did not consider post-operative urinary retention (POUR) as a primary outcome. The purpose of this manuscript is to review the association between glycopyrrolate administration and post-operative urinary retention in the perioperative setting.

## Introduction and background

Postoperative urinary retention (POUR) is defined as the inability to initiate micturition in the presence of a full bladder during the early post-operative period [1]. The reported incidence of POUR in the literature ranges from 2-50% [1]. POUR has been a drawback related to surgical procedures necessitating non-depolarizing neuromuscular blockade (NDNMB) and remains a barrier to providing same day surgical care [2]. The administration of glycopyrrolate in general anesthesia significantly increases the risk of post-operative urinary retention, thus placing an emotional tax on patients as well as an economic burden to the healthcare system.

## Review

Risk Factors for POUR

A meta-analysis conducted by Mason et al. established several risk factors for post-operative urinary retention in patients undergoing ambulatory surgery [3]. Increased age was shown to be associated with POUR (odds ratio of 2.11, 95% confidence interval (CI) 1.15-3.86) in patients older than 60 years old [3]. The presence of lower urinary tract symptoms, such as frequency, urgency, straining, and weak stream, significantly increased the risk of POUR (odds ratio of 2.83, CI 1.57-5.08) (table 1) [3]. Sex was not associated with developing urinary retention post-operatively, although it is generally understood that male sex is a risk factor for non-operative urinary retention due to prostatic hypertrophy [3]. The usage of a preoperative alpha-blocker to treat prostatic hypertrophy significantly decreases the incidence of POUR (odds ratio of 0.37, CI 0.15-0.91) [3].

**Table 1:** Risk Factors for POUR POUR: post-operative urinary retention

Table 1. Risk Factors for POUR
Age >60 [3]
IV administration [1]
Previous Lower Urinary Tract Symptoms [3]
Frequency
Urgency
Straining
Weak Stream
Medications
Anticholinergics (atropine, glycopyrrolate) [4-6]
Beta-blockers [4]
Sympathomimetics [4,5]

Specific surgical procedures affect the incidence of post-operative urinary retention more than others, with the greatest incidence occurring in patients undergoing joint arthroplasty [1]. The incidence of acute POUR following total joint arthroplasty ranges from 0% to 75%, compared to 1-52% for anorectal surgery, and 5.9-38% for hernia repair [1] (Table 2). Patients with comorbidities such as neurologic diseases (stroke, multiple sclerosis, poliomyelitis, spinal lesions, cerebral palsy, and diabetic and alcoholic neuropathy) are at a greater risk for developing urinary retention (Table 3) [4,7]. Medications such as anticholinergics, ß-blockers, and sympathomimetics increase the risk of urinary retention [4-6]. Intravenous administration of more than 750 ml of fluids during the perioperative period also increased the risk of POUR by 2.3 times in patients undergoing hernia repair and anorectal surgery, compared to other surgeries [1].

**Table 2:** Surgical Risk Factors for POUR POUR: post-operative urinary retention

Table 2. Surgical Risk Factors for POUR [1]
Joint arthroplasty
Anorectal surgery
Hernia repair
Previous pelvic surgery

**Table 3:** Comorbid Conditions for POUR POUR: post-operative urinary retention

Table 3. Comorbid Conditions for POUR [4,7]
Stroke
Poliomyelitis
Cerebral Palsy
Multiple Sclerosis
Spinal Lesions
Diabetic Neuropathy
Alcoholic Neuropathy

Glycopyrrolate

Anticholinergics are used in general anesthesia to prevent the muscarinic effect of anticholinesterases, i.e. bradycardia. Glycopyrrolate, an anticholinergic agent, is commonly used in conjunction with neostigmine, an anticholinesterase, for the reversal of neuromuscular blockade created by NDNMB. Bladder detrusor contraction and internal urethral sphincter relaxation during micturition are controlled by parasympathetic stimulation via muscarinic receptors; therefore, blockade of these post-junctional excitatory muscarinic receptors in the detrusor muscle causes bladder hypotonia and increases the frequency of urinary retention [8]. Besides reversing neuromuscular blockade, glycopyrrolate is also used to decrease oral, tracheobronchial, and pharyngeal secretions, and to treat bradycardia [9].

In one of the few available contemporary prospective studies regarding the use of glycopyrrolate, Scott et al. found that glycopyrrolate administration was independently associated with POUR (OR 3.48, CI 1.08-11.24 p = 0.0370) [6]. This study, however, was limited by incomplete data, a low number of patients with both POUR and glycopyrrolate administration, and a low incidence of other risk factors [6]. Additional prospective studies consisting of a larger patient sample should be conducted to obtain a more accurate estimation of the sample population and improve statistical models.

Physical and Psychological Impact

POUR negatively impacts the patient both physically and psychologically. The sense of urinary urgency begins when bladder volume reaches 300 mL [1 ]. An overextended bladder is extremely painful and can cause vomiting, arrhythmias, hypotension or hypertension in some patients [10]. Acute urinary retention may lead to bladder tissue damage and urinary tract infection, impairing renal glomerular and tubular function [11]. POUR is usually managed with urinary catheterization which not only is uncomfortable for the patient, but increases both the risk of bleeding due to trauma in the urogenital tract and urinary tract infections [12]. Patients with catheters in situ that are managed in an outpatient setting are at a 5% per day risk of developing a urinary tract infection, with 2-4% of those further progressing to bacteremia [13,14] Psychologically, patients may also experience great distress due to an unexpected surgical complication as well as embarrassment, vulnerability, and shame of urinary catheterization in non-private settings [15].

Economic Impact

Direct economic impacts from POUR are transferred to the healthcare system. Approximately 28.6 million ambulatory surgery visits were made in the United States in 2010 [16]. Considering that 33% of all procedures were performed on patients aged 65 and over, POUR is a significant barrier to early or same-day discharge in this population [16,17]. It is believed to increase the discharge time in 19% of outpatients [18,19] and is responsible for 20-25% of unplanned inpatient admissions following day-case surgery [20,21]. These unplanned readmissions have direct cost implications for the healthcare organization. Overnight admissions decrease the availability of beds for emergency and elective admissions and results in the loss of 11-70% of the cost savings expected from ambulatory surgery [22]. Some patients may be required to leave the hospital with an indwelling catheter, while also requiring ongoing outpatient management in urology and specialty clinics.

Future Directions

Fortunately, options besides glycopyrrolate exist for reversing NDNMB paralysis so that POUR can be avoided in high-risk patients. Specifically, Sugammadex, a selective relaxant binding agent, is a viable alternative that has recently been introduced into clinical practice [5]. Sugammadex is used to reverse muscle relaxation through strong, rapid, one-to-one, encapsulation with NDNMB in the plasma [6,8]. It has no effect on acetylcholinesterase and prevents blockade of the neuromuscular junction without the undesired muscarinic side effects [22, 23]. Although the direct cost of Sugammadex may be more expensive than glycopyrrolate, it indirectly may be more cost-effective in the long-run due to the amount of time saved by rapid reversal of paralysis as well as reducing incidence of POUR [23].

Cha et al. compared the incidence of urinary retention between patients who received Sugammadex and those who received anticholinesterases with anticholinergics for reversal of neuromuscular blockade in a retrospective cohort. They found that the incidence of urinary retention was significantly lower in the Sugammadex group compared to the glycopyrrolate group (36.1 vs. 48.8%, P = 0.003) [8]. They concluded that the use of Sugammadex was associated with a lower incidence of urinary retention by avoiding glycopyrrolate. Their sample, however, was limited to subjects who underwent total knee arthroplasty, which limited generalizability of their findings. Further prospective studies should be conducted to investigate the role of Sugammadex in reducing the incidence of urinary retention compared to glycopyrrolate administration.

## Conclusions

The number of ambulatory surgical procedures is increasing especially among the elderly population and POUR is one of the most common causes of unplanned hospital admissions after surgery in this patient population. It is, therefore, critical to quantify the incidence of urinary retention and to understand risk factors. This information can be used to optimize operative planning and to help provide better counsel for high-risk patients. Initiating prophylactic interventions such as administration of alpha blockers, magnesium (reduces bladder spasm), and reduction of opioids may decrease the incidence of POUR and should be considered. However, additional randomized controlled trials are necessary to determine the effectiveness of these interventions in the setting of glycopyrrolate administration.

## References

[1] Baldini G, Bagry H, Aprikian A, Carli F (2009). Postoperative urinary retention: anesthetic and perioperative considerations. Anesthesiology.

[2] Salvati EP, Kleckner MS (1957). Urinary retention in anorectal and colonic surgery. Am J Surg.

[3] Mason SE, Scott AJ, Mayer E, Purkayastha S (2016). Patient-related risk factors for urinary retention following ambulatory general surgery: a systematic review and meta-analysis. Am J Surg.

[4] Tammela T, Kontturi M, Lukkarinen O (1986). Postoperative urinary retention. I. Incidence and predisposing factors. Scand J Urol Nephrol.

[5] Petros JG, Rimm EB, Robillard RJ, Argy O (1991). Factors influencing postoperative urinary retention in patients undergoing elective inguinal herniorrhaphy. Am J Surg.

[6] Scott AJ, Mason SE, Langdon AJ (2018). Prospective risk factor analysis for the development of post-operative urinary retention following ambulatory general surgery. World J Surg.

[7] Toyonaga T, Matsushima M, Sogawa N (2006). Postoperative urinary retention after surgery for benign anorectal disease: potential risk factors and strategy for prevention. Int J Colorectal Dis.

[8] Cha J-E, Park SW, Choi YI (2018). Sugammadex use can decrease the incidence of post-operative urinary retention by avoiding anticholinergics: a retrospective study. Anesth Pain Med.

[9] Mier RJ, Bachrach SJ, Lakin RC, Barker T, Childs J, Moran M (2000). Treatment of sialorrhea With glycopyrrolate: a double-blind, dose-ranging study. Arch Pediatr Adolesc Med.

[10] Kamphuis ET, Ionescu TI, Kuipers PW, de Gier J, van Venrooij GE, Boon TA (1998). Recovery of storage and emptying functions of the urinary bladder after spinal anesthesia with lidocaine and with bupivacaine in men. Anesthesiology.

[11] Mustonen S, Ala-Houhala I, Tammela TL (1999). Proteinuria and renal function during and after acute urinary retention. J Urol.

[12] Igawa Y, Wyndaele J-J, Nishizawa O (2008). Catheterization: possible complications and their prevention and treatment. Int J Urol.

[13] Maki DG, Tambyah PA (2001). Engineering out the risk for infection with urinary catheters. Emerg Infect Dis.

[14] Stamm WE (1991). Catheter-associated urinary tract infections: epidemiology, pathogenesis, and prevention. Am J Med.

[15] Wilde MH (2003). Life with an indwelling urinary catheter: the dialectic of stigma and acceptance. Qual Health Res.

[16] Hall MJ, Schwartzman A, Zhang J, Liu X (2017). Ambulatory surgery data from hospitals and ambulatory surgery centers: United States, 2010. Natl Health Stat Report.

[17] Ziemba-Davis M, Nielson M, Kraus K, Duncan N, Nayyar N, Meneghini RM (2019). Identifiable risk factors to minimize postoperative urinary retention in modern outpatient rapid recovery total. Joint Arthroplasty. J Arthroplasty.

[18] Chung F (1995). Recovery pattern and home-readiness after ambulatory surgery. Anesth Analg.

[19] Pavlin DJ, Rapp SE, Polissar NL, Malmgren JA, Koerschgen M, Keyes H (1998). Factors affecting discharge time in adult outpatients. Anesth Analg.

[20] Lau H, Brooks DC (2001). Predictive factors for unanticipated admissions after ambulatory laparoscopic cholecystectomy. Arch Surg.

[21] Awan FN, Zulkifli MS, Mc Cormack O, Manzoor T, Ravi N, Mehigan B, Reynolds JV (2013). Factors involved in unplanned admissions from general surgical day-care in a modern protected facility. Ir Med J.

[22] Naguib M (2007). Sugammadex: another milestone in clinical neuromuscular pharmacology. Anesth Analg.

[23] Paton F, Paulden M, Chambers D (2010). Sugammadex compared with neostigmine/glycopyrrolate for routine reversal of neuromuscular block: a systematic review and economic evaluation† †The research material on which this article is based is protected by Crown Copyright. Br J Anaesth.

